# Comparison of correction factor for both dynamic total thermal insulation and evaporative resistance between ISO 7933 and ISO 9920

**DOI:** 10.1186/s40101-020-00235-9

**Published:** 2020-08-24

**Authors:** Satoru Ueno

**Affiliations:** Japan Organization of Occupational Health and Safety, National Institute of Occupational Safety and Health, 6-21-1 Nagao, Tama-ku, Kawasaki, Kanagawa 214-8585 Japan

**Keywords:** Predicted heat strain (PHS), Thermal insulation, Evaporative resistance, Correction factor

## Abstract

**Background:**

Thermal insulation and evaporative resistance of clothing are the physical parameters to quantify heat transfer and evaporative dissipation from the human body to the environment, respectively. Wind and body movement decrease thermal insulation and evaporative resistance of clothing, which is represented as correction factors for dynamic total thermal insulation (CF_i_) and evaporative resistance (CF_e_), respectively. Then, CF_i_ and CF_e_ are parts of the key parameters to predict heat strain of workers by computer simulation. The objective of this study was to elucidate the difference of CF_i_ and CF_e_ between ISO 7933 and ISO 9920 and compare the difference of predicted rectal temperature, water loss, and exposure time limit calculated by using each correction factor.

**Methods:**

CF_i_ of ISO 7933 (CF_i7933_) and ISO 9920 (CF_i9920_), and CF_e_ of ISO 7933 (CF_e7933_) and two kinds of CF_e_ of ISO 9920 (CF_e9920a_, CF_e9920b_) were compared in terms of relative air velocity, walking speed for three kinds of thermal insulation of clothing. Next, two modified predicted heat strain (PHS) models were developed: modified PHS integrated with CF_i9920_ and CF_e9920a_ (PHS_mA_) and modified PHS integrated with CF_i9920_ and CF_e9920b_ (PHS_mB_). We calculated the rectal temperature, water loss, and exposure time limit by PHS, PHS_mA_, and PHS_mB_ and compared the results.

**Results:**

CF_i7933_ and CF_i9920_ were almost similar in terms of V_ar_ and walking speed, while CF_e9920a_ and CF_e9920b_ were larger than CF_e7933_ when V_ar_ was more than 1.0 m·s^−1^. Intrinsic clothing insulation (I_cl_) diminished the effects of V_ar_ on CF_i7933_, CF_i9920_, CF_e7933_, and CF_e9920b_. However, CF_e9920a_ was not influenced by I_cl_. The predicted rectal temperature and water loss difference were larger between PHS and PHS_mA_ as CF_e_ difference got larger. The duration time when limit of rectal temperature of 38 °C was reached (D_limTre38_) calculated by PHS was significantly longer than PHS_mA_, PHS_mB_ at higher V_ar_.

**Conclusions:**

Precise correction factors for evaporative resistance are required to predict rectal temperature, water loss, and work-time limits.

## Background

Clothing decreases heat transfer between the human body and the environment through convection, conduction, radiation, and evaporation. The thermal characteristics of clothing are mainly represented by total thermal insulation (I_T_) and evaporative resistance (R_eT_). Thermal insulation and the evaporative resistance of clothing depend not only on the clothing itself (fabric and design, such as apertures or folds in the clothing), but also on several conditions, such as the wearer’s activity level, relative air velocity (V_ar_) applied to the wearer, and posture [1–5]. Air flow promotes heat transfer by increased air permeation through the clothing fabric and openings inside the clothing. Human subject experiment using an ergometer showed that forced draft decreased body core temperature and sweat rate by increasing evaporative heat loss [6, 7]. A wearer’s activity also increases heat transfer by the exchange of air between the inside of the clothing and the environment by “pumping effects” [8] through openings in the clothing. The walking movement of thermal manikin facilitated heat dissipation by convection and evaporation. Experimental results of mean skin and core temperature of a walking manikin were closer to a walking human trial data than a standing manikin [9]. When sweat absorbs heat from the skin, it turns into vapor. Thereafter, vapor is transferred to the environment to a greater extent through higher ventilation due to air flow or a wearer’s activity. Increased vapor transfer to the environment decreases the microenvironmental humidity inside the clothing, which promotes evaporation on wetted skin, leading to increased heat transfer between the body and the environment. In a hot environment, the avenue of heat transfer from the human body to the environment is mainly through the evaporation of sweat due to decreased heat transfer by convection from decreased temperature difference between the skin and the environment. When heat loss by sweating is suppressed by vapor-impermeable clothing, though it serves to protect the human body from hazardous materials, thermal physiological strain increases [10–12].

I_T_ and R_eT_ in dynamic conditions are required to calculate heat transfer to the environment and to predict core temperature, water loss, or skin temperature, etc., in thermal models. However, the limited availability of climate chamber, sweating thermal manikin with active simulation, and laminar air flow make it difficult to measure thermal insulation and evaporative resistance of clothing under various specific conditions for air velocity or activity. Then, to numerically predict I_T_ and R_eT_ under wind or active conditions from static conditions, some studies on correction factors for dynamic total thermal insulation (CF_i_) [1, 13–20] and evaporative resistance (CF_e_) [2, 15, 16, 21] have been carried out.

Havenith et al. [1] investigated the effects of both walking and wind on the insulation value of clothing ensembles by conducting human subject experiment. Nilsson and Holmér [13] evaluated the total insulation in the wind and by walking via a thermal manikin. The equations for CF_i_ and CF_e_ in ISO 7933 [22] were developed as part of the BIOMED EU-project led by Malchaire. Three papers [14, 15, 21] were produced in the project. By utilizing the data of Havenith et al. [1] and Nilsson and Holmér [13], Holmér et al. [14] proposed CF_i_ for over 0.6 clo of clothing insulation (Eq. 30 in [14]) and nude (Eq. 29 in [14]) under walking and wind. CF_i_ for nude in [14] was later changed to Eq. 3 in [16] by Havenith et al. Havenith and Nilsson [21] proposed the equations for CF_e_ from the empirical relation of change in *i*_*m*_ with change in heat resistance. Parsons et al. [15] summarized the results and proposed the computer code. Equation 30 [14] and Eq. 3 [16] were included in the present ISO 7933 [22]. In the revision of ISO 7933 [23], predicted heat strain (PHS) model was proposed by Malchaire et al. [24] and adopted in the International Organization for Standardization (ISO) as ISO 7933 [22]. PHS was validated using laboratory (672 experiments) and field (237 experiments) data [25]. Following the publication of ISO 7933 [22], the ISO committee ISO TC159/SC5/WG1 started the ISO 9920 [26] revision. For this work, Havenith [17, 18] reanalyzed more data from a cooperation between Nilsson and Havenith, from Kim and McCullough [19], and from Nilsson et al. [20] on CF_i_ in addition to Holmér et al. [14]. CF_e_ in ISO 9920 [27] was also changed from that of ISO 7933 [22]. By analyzing a more extensive data from Havenith laboratory compared to what was used in ISO 7933 [22], it was found that CF_e_ of ISO 7933 [22] seemed to have overestimated the effects of movement and wind on vapor resistance. After discussions in ISO TC159/SC5/WG1 and presentation of the data, CF_e_ was revised to that of the present ISO 9920 [28]. For static conditions, the Lewis relation and static moisture permeability index (*i*_mst_) were used to estimate evaporative resistance from thermal insulation for both ISO 7933 [22] and ISO 9920 [27]. For dynamic conditions, CF_e_ was derived from the empirical relation between CF_i_ and dynamic moisture permeability index (*i*_mdyn_) in ISO 7933 [22]. On the other hand, in ISO 9920 [27] two equations were provided to calculate CF_e_: one was an empirical equation including relative air velocity and walking speed, and the other was an empirical relation including CF_i_.

The key predictions of PHS are rectal temperature and sweat rate. Some researchers evaluated the predictions of PHS by comparing with physiological data. Kampmann et al. showed a pronounced underestimation of rectal temperature and correct estimation of sweat rate in moderate activity [29]. Parsons [30] pointed out that applying PHS in the assessment of rapidly changing environments and short exposures was not possible. Lundgren et al. also showed that intermittent work exposure challenged the accuracy of the PHS model [31]. They provided the data on the overestimation of PHS simulation on rectal temperature in heavy activity and cooling effect of sweating in recovery [30]. Wang et al. [32] demonstrated that rectal temperature and sweat rate predicted by PHS were higher than those of human subject data when wearing higher thermal insulating or higher evaporative resistance clothing than the scope of PHS model. From the perspective of occupational hygiene in terms of thermal environment, it is important to determine the maximum allowable exposure duration. In ISO 7933 [22], a maximum allowable exposure time is provided based on rectal temperature reaching 38 °C or a cumulative sweat loss limit based on acclimation state. In the determination of the duration time limit, environmental conditions, metabolic rates, and thermal characteristics of clothing should be inputted as important factors. CF_i_ and CF_e_ also play an important role in predicting heat strain under dynamic conditions. It was reported that CF_e_ of ISO 7933 [22] and ISO 9920 [27] were different, and the CF_e_ difference predicted the duration time limit difference for exposure [33]. To predict a suitable CF_e_, it was necessary to further compare among ISO 7933 [22], two kinds of ISO 9920 [27], and experimental data.

The purpose of this paper was to compare the two kinds of CF_i_ or three kinds of CF_e_ for ISO 7933 [22] and ISO 9920 [27] with experimental results and to study the effect of differences in CF_i_ or CF_e_ on predicted rectal temperature, cumulative water loss, and duration time limit of exposure under constant conditions.

## Methods

### Correction factor

Two kinds of CF_i_ (CF_i7933_: Eq. 1.1–3 and CF_i9920_: Eq. 2.1–3) and three kinds of CF_e_ (CF_e7933_: Eq. 3, CF_e9920a_: Eq. 4, and CF_e9920b_: Eq. 5) were summarized in Table 1. There were three functions of CF_i_ in terms of walking speed and relative air velocity according to the intrinsic thermal insulation of clothing (I_cl_). Relative air velocity changes depending on conditions in ISO 7933 [22]: When the data on walking speed and walking direction to wind are provided, relative air velocity is calculated as the VECTOR difference between air velocity and walking speed. When walking direction is unknown, relative air velocity is air velocity if air velocity is larger than walking speed and otherwise walking speed. When both the direction of walking and walking speed were not known, relative air velocity is supposed as air velocity. To avoid complexity in the calculation of relative air velocity from air velocity or walking speed, relative air velocity was directly used to calculate CF_i_ or CF_e_ in this paper. To predict static total water vapor resistance (R_eT_) from static total thermal insulation (I_T_), *i*_mst_ and Lewis relation were applied in ISO 7933 [22] and ISO 9920 [27]. In our calculation, *i*_mst_ of 0.38 was used as normal clothing. Here, walking included only the effect of body movement by walking, excluding the effect of air velocity due to walking. First, we compared the independent effects of relative air velocity and walking speed between CF_i7933_ and CF_i9920_. Table 2 shows the validity of ISO 7933 [22] and ISO 9920 [27] concerning relative air velocity, walking speed, and I_cl_. First, when we calculated the effect of relative air velocity on CF_i_, walking speed was fixed at 0.01 m·s^−1^ and relative air velocity varied from 0.0 to 3.0 m·s^−1^. For walking speed effect, relative air velocity was fixed at 0.15 m·s^−1^ and walking speed varied from 0.0 to 1.2 m·s^−1^. Second, we similarly compared the independent effect of relative air velocity and walking speed among CF_e7933_, CF_e9920a_, and CF_e9929b_. Third, we compared the combined effects of relative air velocity and walking speed on CF_i_ or CF_e_. The relative value of CF_i9920_ to CF_i7933_, CF_e9920a_ to CF_e7933_, and CF_e9920b_ to CF_e7933_ was calculated for a two-dimensional area of relative air velocity from 0.0 to 3.0 m·s^−1^ and walking speed from 0.0 to 1.2 m·s^−1^.

**Table 1:** Correction factors for dynamic total thermal insulation (CF_i_) and evaporative resistance (CF_e_) of ISO 7933 [22] and ISO 9920 [27]

Correction factor	I_cl_	ISO7933 [22]	ISO9920 [27]
CF_i_	Nude	Exp (− 0.472 V_ar_ + 0.047 V_ar_^2^ − 0.342 V_w_ + 0.117 V_w_^2^) (= CorrI_a_) …(1.1)	Exp (− 0.533 × (V_ar_ − 0.15) + 0.069 × (V_ar_ − 0.15)^2^ − 0.462 V_w_ + 0.201 V_w_^2^) (= CorrI_a_) …(2.1)
	0 < I_cl_ < 0.6	((0.6 − I_cl_) × CorrI_a_ + I_cl_ × CorrI_T_)/0.6 …(1.2)	((0.6 − I_cl_ ) × CorrI_a_ × I_a_ + I_cl_ × CorrI_T_ × I_T_(0.6)) / (0.6 × I_T_(I_cl_)) …(2.2)
	I_cl_ ≥ 0.6	Exp (0.043 − 0.398 V_ar_ + 0.066 V_ar_^2^ − 0.378 V_w_ + 0.094 V_w_^2^) (= CorrI_T_) …(1.3)	Exp (− 0.281 × (V_ar_ − 0.15) + 0.044 × (V_ar_ − 0.15)^2^ − 0.492 V_w_ + 0.176 V_w_^2^) (= CorrI_T_) …(2.3)
CF_e_	All range	CorrI_T_/(2.6 CorrI_T_^2^ − 6.5 CorrI_T_ + 4.9) …(3) (If i_mdyn_ = i_mst_ × (2.6 × CorrI_T_^2^ − 6.5 × CorrI_T_ + 4.9) > 0.9, then i_mdyn_ = 0.9)	Exp (− 0.468 × (V_ar_ − 0.15) + 0.080 × (V_ar_ − 0.15)^2^ − 0.874 V_w_ + 0.358 V_w_^2^) …(4) 0.3 − 0.5 × CorrI_T_ + 1.2 × CorrI_T_^2^ …(5)

*I*_*a*_ thermal insulation of air layer in nude, *I*_*cl*_ intrinsic thermal insulation of clothing, *V*_*ar*_ relative air velocity, *V*_*w*_ walking speed, *I*_*T*_*(0.6)* static total thermal insulation at 0.6 clo, *I*_*T*_*(I*_*cl*_*)* static total thermal insulation at I_cl_, *CorrI*_*a*_ correction factors for dynamic thermal insulation in nude, *CorrI*_*T*_ correction factors for dynamic total thermal insulation over 0.6 clo of I_cl_, *i*_*mst*_ static moisture permeability index, *i*_*mdyn*_ dynamic moisture permeability index

**Table 2:** Ranges of validity for ISO 7933 [22] and ISO 9920 [27]

Parameter	ISO 7933 [14]	ISO 9920 [27]
T_a_	15–50 °C	-
T_r_–T_a_	0–60 °C	-
M	52–232 W·m^−2^	-
V_ar_	0.0–3.0 m·s^−1^	0.15–3.50 m·s^−1^
V_w_	0.0–1.5 m·s^−1^	0.0–1.2 m·s^−1^
I_cl_	0.1–1.0 clo	0.0–1.4 clo

*T*_*a*_ air temperature, *T*_*r*_ mean radiant temperature, *M* metabolic rate, *V*_*ar*_ relative air velocity, *V*_*w*_ walking speed, *I*_*cl*_ intrinsic thermal insulation of clothing

### Integration of CF_i9920_ and CF_e9920a_ or CF_e9920b_ to PHS

To investigate the effect of different correction factors on PHS, two modified PHS (PHS_mA_, PHS_mB_) were developed: PHS_mA_ with CF_i9920_ and CF_e9920a_, and PHS_mB_ with CF_i9920_ and CF_e9920b_. First, we calculated the difference of final rectal temperature and water loss calculated between PHS and PHS_mA_ under condition A, condition B, condition C, and condition D (Table 3) with a maximum calculation time of 1 h. In this study, we supposed continuous work for 1 h without taking a break. Other calculation conditions are presented in Table 3: moderate metabolic rate (145 W·m^−2^), height (1.70 m), weight (65 kg), drink available, acclimated, *i*_mst_ (0.38), relative humidity (RH) (from 0 to 100%), and ambient temperature (from 30 to 40 °C). Moderate metabolic rate corresponds to sustained hand and arm work (hammering nails, filing) and arm and leg work (off-road operation of lorries, tractors, or construction equipment) [22]. Furthermore, 1.0 clo (1 clo, 0.155 m^2^·°C·W^−1^) of I_cl_ was used because 1.0 clo was the maximum thermal insulation of clothing, as shown in Table 2. Next, duration time when limit of rectal temperature of 38 °C was reached (D_limTre38_) was compared between PHS_mA_ and PHS and between PHS_mA_ and PHS_mB_ at four conditions of relative air velocity and walking speed (conditions A, B, C, D) with a maximum calculation time of 8 h. Paired *t* test was used to test the significant difference between each model. Here, we supposed a worst-case scenario: continuous work for 8 h without taking a break. The total number of plots was 847, where RH varied from 0 to 100% at 10% intervals, ambient temperature from 30 to 40 °C at 1 °C intervals and mean radiant temperature from ambient temperature to ambient temperature + 60 °C at 10 °C intervals. The other calculation conditions of metabolic rate, height, weight, heat acclimation, *i*_mst_ value, and drink availability are the same as listed in Table 3.

**Table 3:** Input parameters of PHS, PHS_mA_ to calculate difference in predicted core temperature and accumulated water loss

Individual condition	V_ar_	V_w_	I_cl_
Condition A	3.0 m·s^−1^	0.1 m·s^−1^	0.3 clo
Condition B	3.0 m·s^−1^	0.1 m·s^−1^	1.0 clo
Condition C	0.15 m·s^−1^	0.1 m·s^−1^	0.3 clo
Condition D	0.15 m·s^−1^	0.1 m·s^−1^	1.0 clo
Common condition	Input parameter		
T_a_	Every 0.1 °C (30–40 °C)		
RH	Every 1% (0–100%)		
M	145 W·m^−2^		
Height	1.70 m		
Weight	65 kg		
Acclimation	Acclimated		
Drink availability	Available		
*i*_mst_	0.38		
Calculation program	PHS, PHS_mA_		
Calculation time	1 h		

*V*_*ar*_ relative air velocity, *V*_*w*_ walking speed, *I*_*cl*_ intrinsic thermal insulation of clothing, *T*_*a*_ air temperature, *RH* relative humidity, *M* metabolic rate, *i*_*mst*_ static moisture permeability index, *PHS* predicted heat strain, *PHS*_*mA*_ modified PHS including equation (2.1–3) of correction factor for dynamic total thermal insulation and Eq. (4) of correction factor for dynamic total evaporative resistance. Equations are shown in Table 1

## Results

**Fig. 1:**
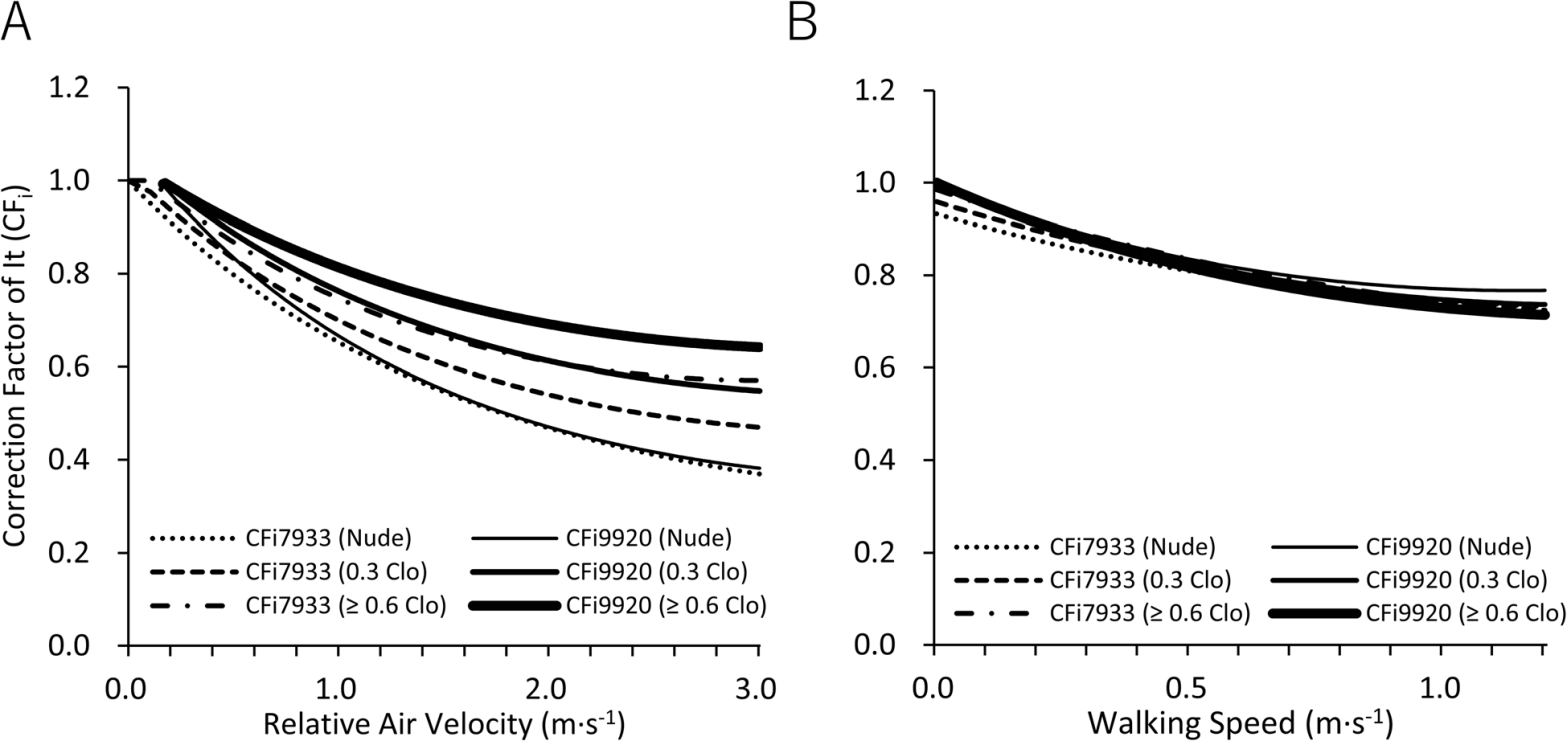
CF_i7933_ and CF_i9920_ in nude, clothing thermal insulation of 0.3 clo and larger than or equal to 0.6 clo in terms of **a** relative air velocity and **b** walking speed

**Fig. 2:**
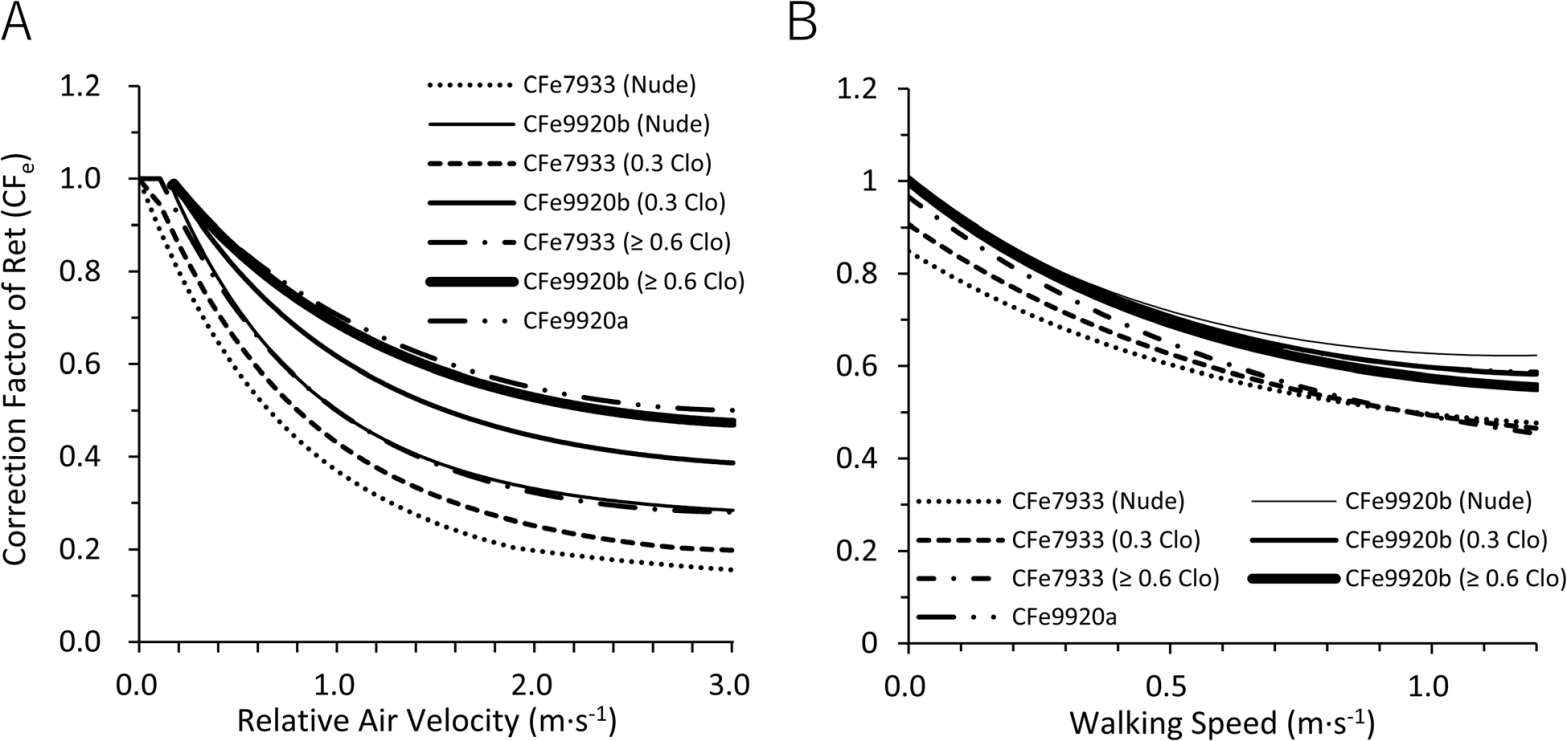
CF_e7933_, CF_e9920a_, and CF_e9920b_ in nude, clothing thermal insulation of 0.3 clo and larger than or equal to 0.6 clo in terms of **a** relative air velocity and **b** walking speed

**Fig. 3:**
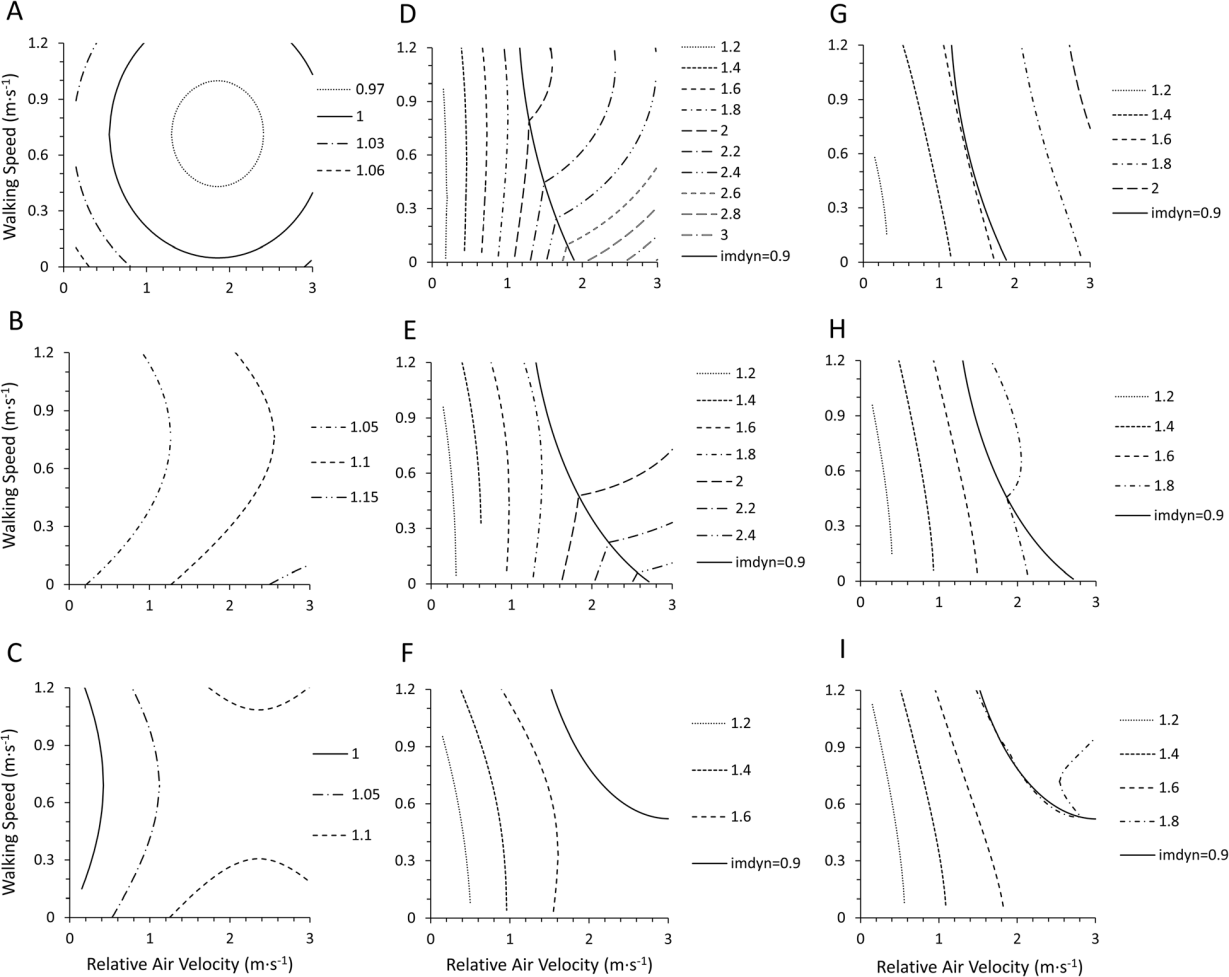
The ratios of CF_i9920_ to CF_i7933_, **a** in nude, **b** clothing thermal insulation of 0.3 clo, **c** clothing thermal insulation larger than or equal to 0.6 clo. The number of lines represents the ratio of CF_i9920_ to CF_i7933_. The ratios of CF_e9920a_ to CF_e7933_, **d** in nude, **e** clothing thermal insulation of 0.3 clo, **f** clothing thermal insulation larger than or equal to 0.6 clo. The number of lines represents the ratio of CF_e9920a_ to CF_e7933_. The ratios of CF_e9920b_ to CF_e7933_, **g** in nude, **h** clothing thermal insulation of 0.3 clo, **i** clothing thermal insulation larger than or equal to 0.6 clo. The number of lines represents the ratio of CF_e9920b_ to CF_e7933_. The line of *i*_mdyn_ of 0.9 is illustrated in the figures

CF_i7933_ and CF_i9920_ decreased similarly with relative air velocity and walking speed in nude and under the clothing thermal insulation of 0.3 clo and more than 0.6 clo (Fig. 1a). The reduction rates of CF_i7933_ and CF_i9920_ for relative air velocity were largest in nude, second in 0.3 clo, and least in larger than 0.6 clo. However, CF_i7933_ and CF_i9920_ did not differ in nude, the clothing thermal insulation of 0.3 clo and more than 0.6 clo in terms of walking speed (Fig. 1b). CF_e7933_ was smaller than CF_e9920a_ and CF_e9920b_ in both for relative air velocity and walking speed. For a nude condition at 3.0 m·s^−1^ of relative air velocity, CF_e9920a_ was larger than CF_e7933_ by more than three times. For larger than 0.6 clo, CF_e9920a_ and CF_e9920b_ were almost the same and were about two times larger than CF_e7933_ (Fig. 2a). For the effect of walking speed, CF_e9920a_ and CF_e9920b_ were also as large as CF_e7933_ (Fig. 2b). We compared the combined effect of relative air velocity and walking speed on CF_i7933_ and CF_i9920_, CF_e7933_ and CF_e9920a_, and CF_e7933_ and CF_e9920b_ in Fig. 3. For CF_i_, the ratio of CF_i9920_ to CF_i7933_ was almost 1 in the large part of the calculated scope (relative air velocity 0.15–3.0 m·s^−1^, walking speed 0.01–1.2 m·s^−1^), indicating that CF_i9920_ and CF_i7933_ were almost the same (Fig. 3a–c). For CF_e_, both the contours of the ratios of CF_e9920a_ and CF_e9920b_ to CF_e7933_ were almost parallel to the y-axis, which means that both CF_e9920a_ and CF_e9920b_ change similarly in terms of walking speed (Fig. 3d–i). The ratio of CF_e9920a_ to CF_e7933_ was the largest in nude (Fig. 3d), next in 0.3 clo (Fig. 3e), and the smallest in more than 0.6 clo (Fig. 3f). The ratio of CF_e9920b_ to CF_e7933_ was not different in terms of clothing thermal insulation in the calculated scope (Fig. 3g–i).

**Fig. 4:**
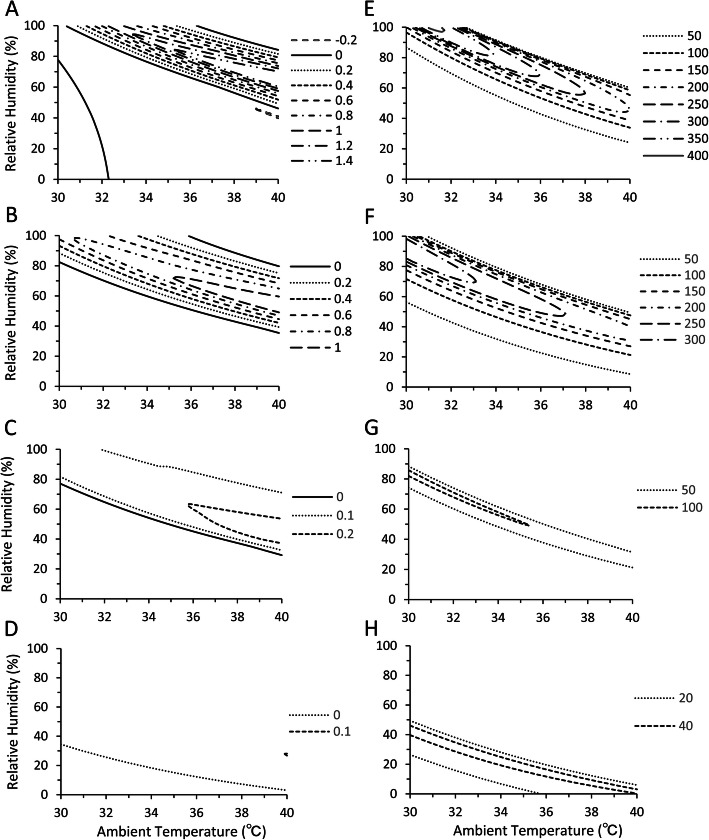
Difference in predicted rectal temperature between PHS and PHS_mA_, **a** under condition A, **b** condition B, **c** condition C, and **d** condition D in Table 3. The units of the line number are degrees centigrade. Difference in predicted water loss between PHS and PHS_mA,_ **e** under condition A, **f** condition B, **g** condition C, and **h** condition D in Table 3. The units of the line number are milliliters

Figure 4 provides the predicted rectal temperature (Fig. 4a–d) and water loss difference (Fig. 4e–h) between PHS and PHS_mA_ for a 1-h exposure with a scope of ambient temperature from 30 to 40 °C and RH from 0 to 100% in four conditions (conditions A–D). Under condition A, in high ambient temperature and RH region, the predicted rectal temperature by PHS_mA_ was higher than that by PHS. The maximum rectal temperature difference in the scope was about 1.4 °C (Fig. 4a). And the maximum water loss difference was about 400 ml (Fig. 4e). The region where the predicted water loss differed was almost the same area as that of the rectal temperature difference (Fig. 4e). Under condition B (Fig. 4b), the region where predicted rectal temperature and water loss differed was similar to condition A. The maximum rectal temperature difference was about 1.0 °C (Fig. 4b) and maximum water loss difference (Fig. 4f) was about 300 ml. Under condition C or D, both rectal temperature and water loss between PHS and PHS_mA_ did not differ as much as condition A or B.

**Fig. 5:**
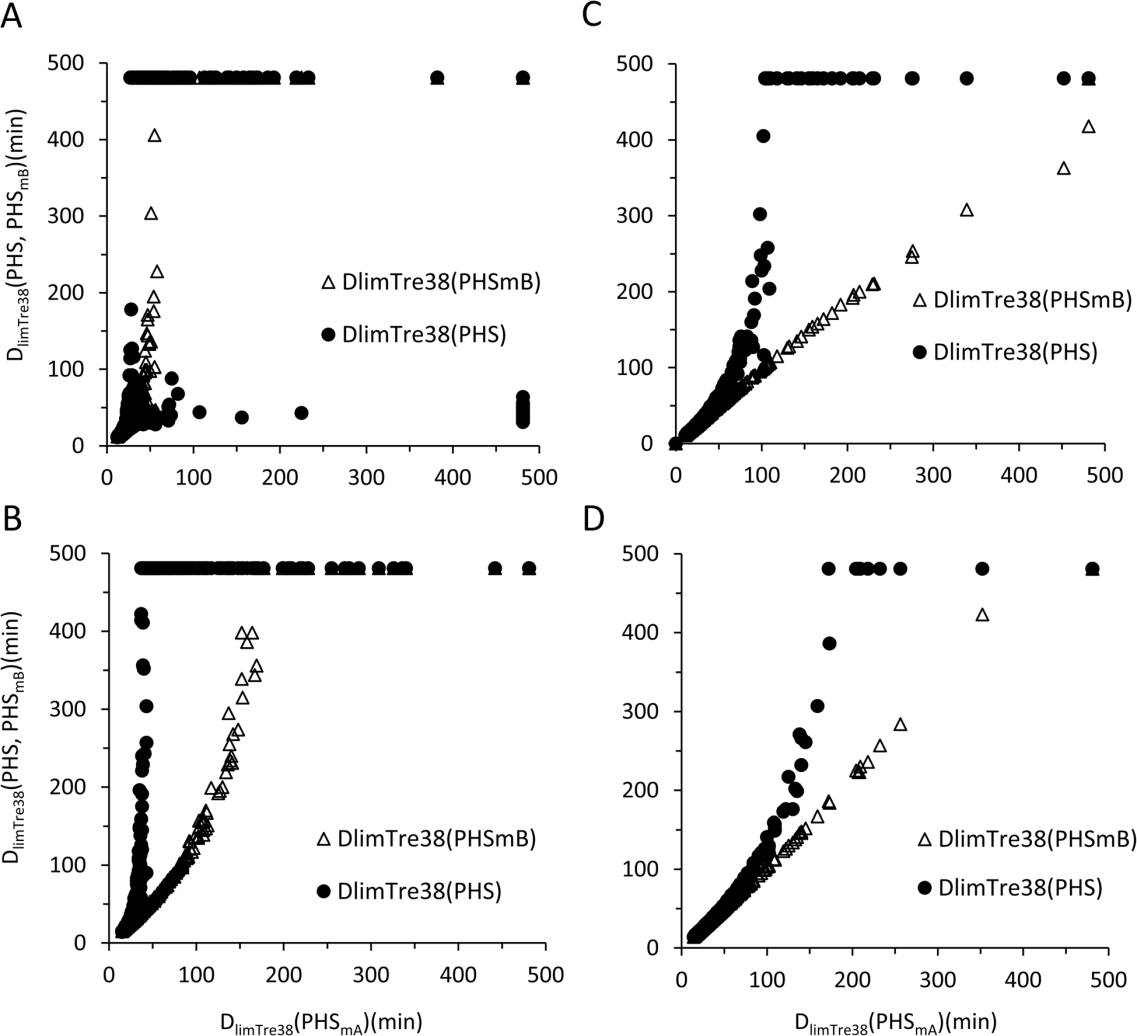
Predicted time until rectal temperature reaches 38.0 °C (D_limTre38_) of PHS, PHS_mA_ and PHS_mB_ under **a** condition A, **b** condition B, **c** condition C, and **d** condition D in Table 3. In each figure, the x axis represents D_limTre38_ by PHS_mA_ and y axis by PHS and PHS_mB_. The number of plots was 121, where relative humidity varies from 0 to 100% at 10% intervals and the ambient temperature from 30 to 40 °C at 1 °C intervals. The maximum calculation time was 480 min

The differences in D_limTre38_ between PHS_mA_ and PHS and between PHS_mA_ and PHS_mB_ are shown in Fig. 5 for the four conditions (conditions A, B, C, and D). D_limTre38_ differences between PHS and PHS_mA_ and between PHS_mA_ and PHS_mB_ were the largest under condition A and second largest under condition B. The largest D_limTre38_ differences between PHS and PHS_mA_ were 454, 444, 377, and 309 min under conditions A, B, C, and D, respectively. The largest D_limTre38_ differences between PHS_mA_ and PHS_mB_ were 434, 310, 89, and 71 min under conditions A, B, C, and D, respectively. D_limTre38_ values of PHS were significantly larger than PHS_mA_ and PHS_mB_ by paired *t* test under the four tested conditions (*P* < 0.001). D_limTre38_ values of PHS_mB_ were significantly larger than PHS_mA_ by paired *t* test under the four tested conditions (*P* < 0.001).

## Discussion

In this study, we compared CF_i_ and CF_e_ between ISO 7933 [22] and ISO 9920 [27]. The vapor resistance value was reduced less in ISO9920 than in ISO7933. CF_i_ was close to each other, but CF_e9920a_ and CF_e9920b_ were larger than CF_e7933_. To the best of our knowledge, there is no other study to compare correction factors of ISO 7933 [22] and ISO 9920 [27] in detail in terms of relative air velocity and walking speed. Next, we investigated the effect of CF_e_ differences on predicted rectal temperature and water loss under warm and humid conditions. When the difference between CF_e7933_ and CF_e9920a_ was large, the predicted rectal temperature and water loss were higher in PHS_mA_ than PHS at high ambient temperature and RH. D_limTre38_ values by PHS were significantly larger than those of PHS_mA_ and PHS_mB_.

**Fig. 6:**
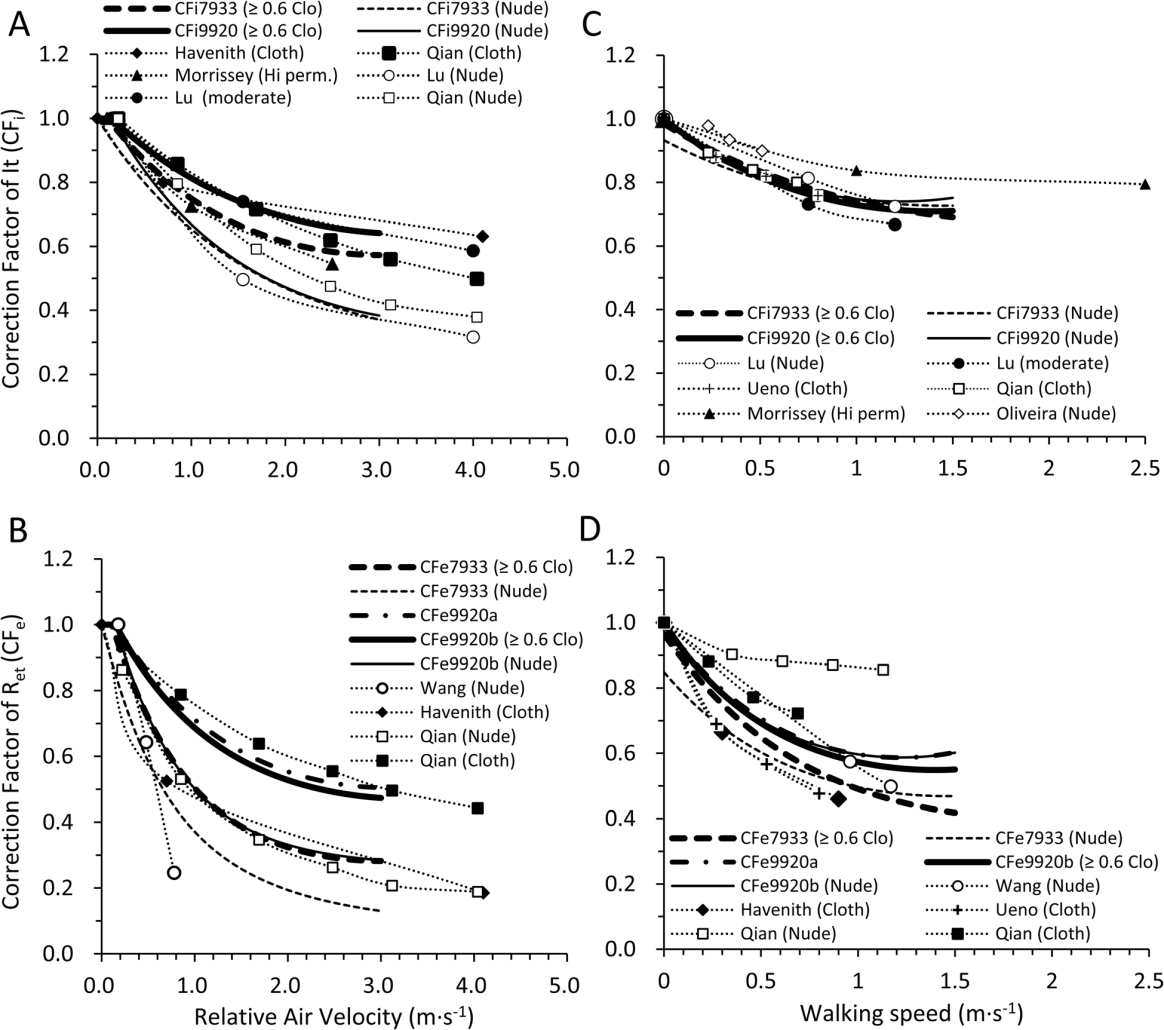
Correction factors of ISO 7933, ISO 9920, and experimental results. **a** Correction factors of I_T_ in terms of relative air velocity, **b** correction factors of R_eT_ in terms of relative air velocity, **c** correction factors of I_T_ in terms of walking speed, and **d** correction factors of R_eT_ in terms of walking speed. The averages of intrinsic thermal insulation of the tested clothing used in the experiments were the following: **a** 1.1 clo (Havenith et al. [1] (cloth)), 0.0 clo (Qian and Fan [34] (nude)), 0.7 clo (Qian and Fan [35] (cloth)), 1.9 clo (Morrissey and Rossi [36] (Hi perm.)), 0.0 clo (Lu et al [37] (nude)), 1.8 clo (Lu et al. [37] (moderate)); **b** 1.1 clo (Havenith et al. [2] (cloth)), 0.0 clo (Qian and Fan [34] (nude)), 0.7 clo (Qian and Fan [35] (cloth)), 0.0 clo (Wang et al. [38] (nude)); **c** 0.0 clo (Lu et al. [37] (nude)), 1.5 clo (Lu et al. [37] (moderate)), 1.2 clo (Ueno and Sawada [39] (Workwear)), 0.7 clo (Qian and Fan [34] (Cloth)), 1.9 clo (Morrissey and Rossi [36] (Hi perm.)), 0.0 clo (Oliveira et al [40] (nude)); and **d** 0.0 clo (Wang et al. [38] (nude)), 1.1 clo (Havenith et al. [2] (cloth)), 1.2 clo (Ueno and Sawada [39] (workwear)), 0.0 clo (Qian and Fan [34] (Nude)), 0.7 clo (Qian and Fan [35] (cloth)). When only the total thermal insulation was given in the reference, the intrinsic thermal insulation was calculated using the equations in ISO 7933

Many researchers [1, 2, 14, 20, 21, 34–40] demonstrated the dependence of CF_i_ or CF_e_ on relative air velocity and walking speed mainly with human subject study or thermal manikin (Fig. 6a–d). In this paper, CF_i_ and CF_e_ of ISO 7933 [22] and ISO 9920 [27] were compared with experimental data including the recent research published after the issuance of ISO 9920 [27]. Concerning CF_i_, CF_i7933_ and CF_i9920_ were close to experimental results both in nude and clothing conditions (Fig. 6a). CF_i_ of Qian (cloth) [35], Morrissey (garment zip fastened) [36] were close to CF_i7933_ (≥ 0.6 clo). Morrissey and Rossi [36] showed that CF_i_ with relative air velocity was lowered in an unfastened garment zip. Thus, how one wears clothing could also influence CF_i_ with relative air velocity. CF_i_ of Lu et al. (nude) [37] was close to those of CF_i7933_ (nude) and CF_i9920_ (nude). CF_i_ of Lu et al. (moderate) [37] was also close to CF_i9920_ (≥ 0.6 clo).

CF_i7933_ and CF_i9920_ in nude conditions decreased with relative air velocity more than in clothing conditions. Qian and Fan [34, 35] and Lu et al. [37] also showed that CF_i_ of a nude body was smaller than a clothed body under the same relative air velocity (Fig. 6a). However, with walking speed, CF_i_ in nude conditions and clothing conditions were almost the same for CF_i7933_ and CF_i9920_ (Fig. 6c). The experimental results also showed that CF_i_ dependence on walking speed was not related to I_cl_ (Fig. 6c).

CF_e_ of experimental data, CF_e7933_, CF_e9920a_, and CF_e9920b_, were different in two ways. First, CF_e9920b_ was larger than CF_e7933_ in both nude and more than 0.6 clo (Fig. 6b, d). CF_e9920a_ was also larger than CF_e7933_. Experiment data of CF_e_ [2, 34, 35, 38, 39] which the standards should be based on were not consistent (Fig. 6b, d). The differences in experimental conditions or calculation methods of R_eT_ in the study of thermal manikin could explain the R_eT_ difference [41]. Second, the CF_e9920a_ did not depend on I_T_. This is contrary to experimental results showing that CF_e_ in nude decreased with relative air velocity to a greater extent than that in clothing. To resolve these discrepancies, more experimental study regarding CF_e_ dependence on relative air velocity and walking speed would be needed.

For the combined effect of relative air velocity and walking speed on CF_i_, Eq. 1.1, 1.2, 2.1, and 2.3 in Table 1 showed that relative air velocity and walking speed independently affected CF_i_. Heat exchange increased in the front trunk (chest, abdomen, pelvis) with a frontal wind. Meanwhile, heat exchange increases more in the arm and foot than the front trunk when walking in nude or light clothing [40]. In a combined condition of wind and walking, the effect of relative air velocity on I_T_ was larger than that of walking speed and affected that of walking speed [40]. To explain these effects, the interaction term of relative air velocity and walking speed could be needed for CF_i_ equation.

A higher CF_e_ induced a smaller maximum evaporation rate and higher wettedness in the skin, leading to a smaller evaporation efficiency. To correct a smaller evaporation efficiency and maintain a balance in heat transfer, the sweat rate increases. In our calculation, PHS_mA_ predicted that water loss increases at a lower ambient temperature than PHS and reached a maximum sweat rate (SW_max_) at lower ambient temperature. In PHS, PHS_mA_ and PHS_mB_, SW_max_ is determined by metabolic rate and acclimation.

SW_max_ = (*M* − 32) × surface area of human body × factor of acclimation (6) where *M* stands for metabolic rate in W·m^−2^ [25]. The surface area of human body was expressed in m^2^. Factor of acclimation was 1.25 for acclimated person and 1.0 for unacclimated person. Before sweat rate reached SW_max_, the rectal temperature did not increase. But after reaching SW_max_, the rectal temperature started to increase in PHS model. Then, the time when the predicted rectal temperature started to increase was almost equivalent to the time when the predicted water loss reached the maximum sweat rate. When predicted sweat rate by PHS_mA_ reaches SW_max_ and by PHS did not, only the predicted rectal temperature by PHS_mA_ increases. This relation explained that the differences in the rectal temperature between PHS and PHS_mA_ were closely related to the differences in predicted water loss. Under every condition, the zone where rectal temperature differed almost overlapped the zone where sweat rate differed (Fig. 4). The difference in rectal temperature and water loss was larger under conditions A or B than conditions C or D. A larger difference in CF_e_ (Table 4) would contribute to a larger difference in predicted rectal temperature and water loss.

**Table 4:** Correction factor for dynamic total thermal insulation and evaporative resistance of PHS, PHS_mA_ and PHS_mB_ for four conditions (conditions A–D)

Individual condition	CF_i_			CF_e_		
	PHS	PHS_mA_	PHS_mB_	PHS	PHS_mA_	PHS_mB_
Condition A	0.46	0.54	0.54	0.18	0.46	0.38
Condition B	0.55	0.61	0.61	0.26	0.46	0.44
Condition C	0.93	0.98	0.98	0.83	0.92	0.97
Condition D	0.95	0.95	0.95	0.88	0.92	0.91

*CF*_*i*_ correction factor for dynamic total thermal insulation, *CF*_*e*_ correction factor for dynamic total evaporative resistance, *PHS* predicted heat strain, *PHS*_*mA*_ modified PHS including CF_i_ of Eqs. (2.1–3) and CF_e_ of Eq. (4), *PHS*_*mB*_ modified PHS including CF_i_ of Eq. (2.1–3) and CF_e_ of Eq. (5). Equations are shown in Table 1

Our study showed that differences of D_limTre38_ between PHS and PHS_mA_ were largest in condition A (Fig. 5). The largest difference in CF_e_ among PHS, PHS_mA_, and PHS_mB_ in condition A (Table 4) could result in Dlim_Tre38_ difference. The large amount of water loss due to a low evaporation efficiency by a high CF_e_ increased the probability of reaching the maximum sweat rate and an elevated rectal temperature. Thus, at high CF_e_, rectal temperature increased in a lower heat stress environment than for a low CF_e_. Under heat stress conditions where body rectal temperature started to increase, an inaccuracy in CF_e_ led to a large prediction error for D_limTre38_ values. Originally, predicting heat strain under such boundary conditions was required to avoid heat disorders. As such, the prediction errors due to an inaccurate CF_e_ should be lowered as much as possible under such boundary conditions. Since many kinds of clothing exist, it could be difficult to develop a CF_e_ that covers all kinds of clothing. Thus, it would be necessary to derive a CF_i_ or CF_e_ that is specialized for work clothing to prevent work-related heat disorders. Many other factors, except for wind or walking activity, such as how clothes fit, posture, and openings, were reported to affect I_T_ [42]. Further study is needed to estimate precise correction factor considering other factors.

The clothing area factor (f_cl_), defined as the ratio of the clothing surface area to the body surface area, also plays an important role in the analysis of heat exchange between a clothed body and the environment. Though f_cl_ is decided only by static clothing thermal insulation, it is used in both static and dynamic conditions in ISO 7933 [22]. Since f_cl_ was shown to depend on clothes’ fit or posture [43] and clothing shape was changed by wind [44], some corrections to f_cl_ should be considered.

Moreover, the scope of relative air velocity is limited to 3.0 and 3.5 m·s^−1^ for ISO 7933 [22] and ISO 9920 [27], respectively. When PHS is applied to outdoor work, the scope should be extended to an air velocity of more than 3.0 m·s^−1^.

## Conclusions

The correction factors for dynamic total thermal insulation (CF_i9920_) and evaporative resistance (CF_e9920a_ and CF_e9920b_) proposed in ISO 9920 [27] were compared with those of ISO 7933 (CF_i7933_ and CF_e7933_) [22]. The vapor resistance value was reduced less in ISO9920 than in ISO7933. CF_i_ were close to each other; however, CF_e_ of ISO 9920 [33] was much larger than that of ISO 7933 [22]. The relation of one CF_e_ in ISO 9920 [27] on relative air velocity was not influenced by the intrinsic thermal insulation of clothing. The difference in CF_e_ lead to a difference in predicted rectal temperature and water loss in the critical region of ambient temperature and RH where predicted sweat rate reached maximum sweat rate. Duration time when limit of rectal temperature of 38 °C is reached (D_limTre38_) was different according to the CF_e_ used in the calculation. A larger difference in CF_e_ results in a larger difference in D_limTre38_ value. The development of a correct CF_e_ is required to predict appropriate work time limits in hot working environments.

## Data Availability

The datasets used and/or software during the current study are available from the corresponding author on reasonable request.

## Abbreviations

D_limTre38_: Duration time when limit of rectal temperature of 38 °C is reached (minutes)
CF_e_: Correction factor for dynamic total evaporative resistance (dimensionless)
CF_e7933_: CF_e_ of ISO 7933 (dimensionless)
CF_e9920a_: CF_e_ of ISO 9920 using relative air velocity and walking speed (dimensionless)
CF_e9920b_: CF_e_ of ISO 9920 using CF_i9920_ (dimensionless)
CF_i_: Correction factor for dynamic total thermal insulation (dimensionless)
CF_i7933_: CF_i_ of ISO 7933 (dimensionless)
CF_i9920_: CF_i_ of ISO 9920 (dimensionless)
ISO: International organization for standardization
I_T_: Total thermal insulation (m^2^·K·W^−1^)
PHS: Predicted heat strain
PHS_mA_: Modified PHS integrated with CF_i9920_ and CF_e9920a_
PHS_mB_: Modified PHS integrated with CF_i9920_ and CF_e9920b_
R_eT_: Total evaporative resistance (m^2^·kPa·W^−1^)
